# *In vitro* large-scale experimental and theoretical studies for the realization of bi-directional brain-prostheses

**DOI:** 10.3389/fncir.2013.00040

**Published:** 2013-03-14

**Authors:** Paolo Bonifazi, Francesco Difato, Paolo Massobrio, Gian L. Breschi, Valentina Pasquale, Timothée Levi, Miri Goldin, Yannick Bornat, Mariateresa Tedesco, Marta Bisio, Sivan Kanner, Ronit Galron, Jacopo Tessadori, Stefano Taverna, Michela Chiappalone

**Affiliations:** ^1^School of Physics and Astronomy, Tel Aviv UniversityTel Aviv, Israel; ^2^Department of Neuroscience and Brain Technologies, Istituto Italiano di TecnologiaGenova, Italy; ^3^Department of Informatics, Bioengineering, Robotics and System Engineering, University of GenovaGenova, Italy; ^4^IMS laboratory, UMR CNRS 5218, University of BordeauxTalence, France; ^5^Department of Neurobiology, George S. Wise Faculty of Life Sciences, Sagol School of Neuroscience, Tel Aviv UniversityTel-Aviv, Israel

**Keywords:** *In vitro* modular networks, whole brain, lesioned circuits, *in silico* neuronal circuit, hardware spiking neural network

## Abstract

Brain-machine interfaces (BMI) were born to control “actions from thoughts” in order to recover motor capability of patients with impaired functional connectivity between the central and peripheral nervous system. The final goal of our studies is the development of a new proof-of-concept BMI—a neuromorphic chip for brain repair—to reproduce the functional organization of a damaged part of the central nervous system. To reach this ambitious goal, we implemented a multidisciplinary “bottom-up” approach in which *in vitro* networks are the paradigm for the development of an *in silico* model to be incorporated into a neuromorphic device. In this paper we present the overall strategy and focus on the different building blocks of our studies: (i) the experimental characterization and modeling of “finite size networks” which represent the smallest and most general self-organized circuits capable of generating spontaneous collective dynamics; (ii) the induction of lesions in neuronal networks and the whole brain preparation with special attention on the impact on the functional organization of the circuits; (iii) the first production of a neuromorphic chip able to implement a real-time model of neuronal networks. A dynamical characterization of the finite size circuits with single cell resolution is provided. A neural network model based on Izhikevich neurons was able to replicate the experimental observations. Changes in the dynamics of the neuronal circuits induced by optical and ischemic lesions are presented respectively for *in vitro* neuronal networks and for a whole brain preparation. Finally the implementation of a neuromorphic chip reproducing the network dynamics in quasi-real time (10 ns precision) is presented.

## Introduction

Millions of people worldwide are affected by neurological disorders that disrupt connections between brain and body, causing paralysis, or impair cognitive capabilities. This number is likely to increase in coming years, yet current assistive technology is still limited. Over the last decade Brain-Machine Interfaces (BMIs) and neuro-prostheses (Nicolelis, 2003; Hochberg et al., 2006, 2012; Nicolelis and Lebedev, 2009) have been the object of extensive research and offer the promise of treatment for such disabilities. These devices could profoundly improve the quality of life for affected individuals, and could have a more widespread impact on society.

Neural interfaces have mainly been devoted to restoring motor function that is lost due to injuries at the level of the spinal cord (Collinger et al., 2013), or to recover sensorial capacities, e.g., artificial retinal or cochlear implants (Chader et al., 2009). However, recent interest has also focused on neural prostheses for restoring cognitive functions. For example, a hippocampal prosthesis for improving memory function in behaving rats was recently presented (Berger et al., 2011, 2012), and the same group has also tested a device in primate prefrontal cortex aimed at restoring impaired cognitive functions (Hampson et al., 2012; Opris et al., 2012).

The realization of such prostheses implies that we know how to interact with neuronal cell assemblies, taking into account the intrinsic spontaneous activation of neuronal networks and understanding how to drive them into a desired state in order to produce a specific behavior. The long-term goal of replacing damaged brain areas with artificial devices requires neural network-like prosthetics or models that could be fed with recorded electrophysiological patterns and that could provide a substitute output to recover the desired functions. While ultimately this approach must be tested and applied *in vivo*, important insights could be gained using *in vitro* systems of increasing architectural complexity, which can be more easily and thoroughly accessed, monitored, manipulated, and modeled than *in vivo* systems (at least at present).

The final goal of the studies presented in this paper is to develop a test-bed for the development of a new generation of neuro-prostheses capable of restoring lost communication between neuronal circuits. These studies constitute the object of the European project BRAIN BOW (www.brainbowproject.eu). Healthy and lesioned *in vitro* neuronal circuits are characterized in parallel to the development of *in silico* neuronal networks, with the goal of establishing bi-directional communication to mimic or bypass an injured neuronal network. In order to develop an experimental and computational platform for the prototyping of neuro-prostheses, we followed a bottom-up approach using *in vitro* biological neuronal systems with increasing structural complexity. Our approach takes advantage of the unique features of *in vitro* neuronal cultures, which represent a powerful experimental model to investigate the inherent properties of neuronal cell assemblies as a complement to artificial computational models. We use engineered networks of increasing structural complexity, from isolated finite-size networks up to interacting assemblies, as a model of intercommunicating neuronal circuitries. Moreover, we scaled our studies up to the isolated whole guinea-pig brain (IWB), to translate to an *in vivo* model.

In this paper we present the overall multidisciplinary strategy and preliminary results on the different building blocks of the project. The structure-function relationship of “finite size circuits” was characterized with single cell resolution by combining calcium imaging and immunocytochemistry. Similarly to what previously observed in isolated neuronal clusters (Shein-Idelson et al., 2010), we found that the frequency of synchronous network events increased with circuit size. This result was reproduced by *in silico* neural network models based on Izhikevich neurons with scale-free connectivity. The feasibility of controlled network lesions was explored by optically transecting cell processes and monitoring the subsequent change in functional network connectivity. In addition, in a whole brain preparation, a focal ischemic lesion in the hippocampus was demonstrated to cause an interruption of the limbic olfactory pathway. Finally, a neural network hardware model with arbitrary connectivity based on Izhikevich neurons, working at nanosecond time scale, is presented. These experimental and computational platforms represent a starting point for restoring functional closed-loop communication in a neuronal network with lesioned circuitries.

## Materials and methods

### Experimental models

**Figure 1:**
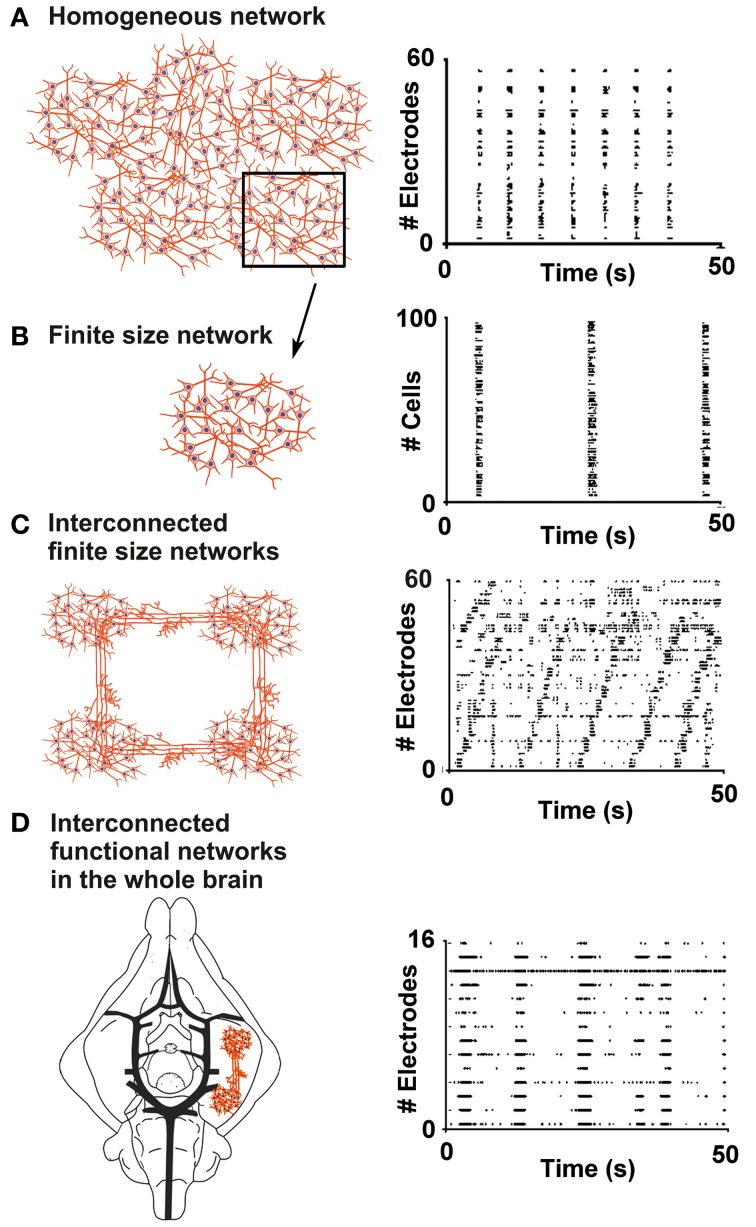
**From finite size networks up to the whole brain: a bottom-up approach. (A)** Sketch of a homogeneous network composed of about 1000 neurons (left panel) and the typical raster plot of its electrophysiological activity, recorded by using 60 electrodes of a Micro Electrode Array (MEA) chip (right panel). The black box highlights a sub-region of the homogeneous network, which can be described as a finite size network (black arrow pointing to panel **B**). **(B)** Sketch of a finite size network used in the framework of this paper, composed of about 100 neurons (left panel), and its raster plot, obtained by calcium imaging recording (right panel). **(C)** Scheme of interconnected finite size networks, each composed of about 100 neurons (left panel), and the raster plot of the electrophysiological activity recorded by a MEA (right panel). **(D)** Sketch of the *in-vitro* whole brain of a guinea pig composed by interconnected functional networks (left panel), and raster plot of the spontaneous periodic events recorded by an array of 16 electrodes (right panel).

The repertoire of activity patterns exhibited by an *in vitro* neural network is strongly dependent on the complexity of its geometry (Shein-Idelson et al., 2011). While homogeneous networks (Figure 1A) tend to display highly stereotyped bursts which spread to most of the connected cells (Kamioka et al., 1996; Van Pelt et al., 2004; Chiappalone et al., 2006; Eytan and Marom, 2006), networks composed of smaller sub-networks with sparse connections (Figure 1C) usually present non-repetitive patterns of sparse spiking and local bursts (Macis et al., 2007; Shein-Idelson et al., 2010). The first cellular model proposed in this work is that of finite size network (Figure 1B), namely an isolated neuronal circuit consisting of a small number of neurons (dozens to a few hundreds) that is still able to spontaneously produce bursts similar to those observed in larger homogeneous networks (cf. section “Results”). Characterization of activity within these assemblies could allow their use as building blocks for larger, more complex structures of interconnecting sub-networks. At the other end of the complexity spectrum we set the isolated whole brain of a guinea pig (Figure 1D). This model is used to investigate the properties of one complex functional neuronal assembly (the olfactory tract, see below) embedded in an intact brain (cf. section “Results”).

#### Finite size networks: patterning, cell culture, and calcium imaging

The procedure adopted for the preparation of “finite size networks” is in accordance with the NIH standards for care and use of laboratory animals and was approved by the Tel-Aviv University Animal Care and Use Committee.

Cultures were prepared as described in Herzog et al. (2011). After the fourth day *in vitro*, the growth medium was enriched with 0.5% Pen-Strep (Biological Industries Beit Haemek), 2% B-27 (Gibco), and 0.75% glutamax (Biological Industries Beit Haemek). Cells were plated at a density of 750 cells/mm^2^ on a 23 mm square glass coverslip previously glued on a 35 mm petri dish. Coverslips were coated with spots of poly-D-lysine (PDL, Sigma), and petri dishes were homogenously coated with PDL. The cells attaching homogeneously on the free surface of the petri dish (i.e., not covered by the glass coverslip) functioned as a “supporting network” (Kleinfeld et al., 1988). PDL spots were created using either manual drop deposition or polydimethylsiloxane (PDMS) stencils. For manual drop deposition, an Eppendorf pipette with a tip of 10 μl capacity was used. The spots were created by touching the tip filled with 2 μl PDL on the coverslip surface and then drying the coverslips at 37°C for 30 min.

When PDMS stencils were used, the procedure to create PDL spots was based on a soft lithography process, as described in Sorkin et al. (2006). Briefly, an SU8-2075 (Micro Chem) mould on a silicon wafer with a feature thickness of approximately 200 μm was used to shape the PDMS. The feature was composed of squares of 700 μm × 700 μm separated by at least 1 mm, in order to obtain isolated neuronal islands. The size of the square was chosen to fit the field of view of a 10× objective in the calcium imaging setup described in detail below and in Herzog et al. (2011). Once the PDMS substrate was shaped and dried on the silicon wafer, the PDMS stencils were detached and placed directly on the glass coverslips. Drops of the PDL solution were dripped onto the PDMS stencil until the features were completely covered. After mild vacuum degassing for 15 min, the excess PDL solution was removed and the sample was dried at 37°C for 30 min. The PDMS stencil was removed before cell plating.

Calcium imaging of the patterned neuronal networks grown on coverslips was performed in buffered-ACSF solution (containing, in mM, 10 HEPES, 4 KCl, 1.5 CaCl_2_, 0.75 MgCl_2_, 139 NaCl, 10 D-glucose, adjusted with sucrose to an osmolarity of 325 mOsm, and with NaOH to a pH of 7.4). In order to load the cells with the calcium-sensitive dye, cultures were incubated for 30 min in 1 ml ACSF supplemented with 1 μl of 10% pluronic acid F-127 (Biotium 59000) and 1 μl Oregon-Green BAPTA-I AM (Invitrogen 06807) previously diluted with 7.6 μl anhydrous-DMSO. Following incubation, cultures were washed with ACSF and recorded at 37°C. In order to avoid artifacts due to evaporation and pH change, the ACSF was replaced every 20 min during the recording session.

Calcium-fluorescence images were acquired with an EMCCD camera (Andor Ixon-885) mounted on an upright Olympus microscope (BX51WI) using a 10× water-immersion objective (Olympus, NA 0.4). Fluorescent excitation was provided via a 120 W mercury lamp (EXFO X-Cite 120PC) coupled to the microscope optical axis with a dichroic mirror, and equipped with an emission filter matching the dye spectrum (Chroma T495LP). Images were acquired at 59 fps in 2 × 2 binning mode using Andor software data-acquisition card (SOLIS) installed on a personal computer.

#### Immunocytochemical staining

At the end of calcium-imaging experiments, cultures were washed twice with PBS, then fixed with 4% PFA (15 min) and left in PBS for not more than 5 days before staining. For immunocytochemical staining, fixed cultures were washed three times with PBS (10 min each) and then incubated with 1% Triton ×100 in PBS for 30 min. Cultures were blocked with 2% BSA, 10% normal serum and 0.5% Triton × 100 in PBS for 2 h at room temperature. The cultures were incubated overnight with the first primary antibody (GAD67, 1:250, Millipore, MAB5406) in blocking solution at 4°C. The next day cultures were incubated with the second primary antibody (MAP2, 1:500, Chemicon, AB5622) overnight at 4°C. Cultures were then washed three times with TBS and incubated with the secondary antibodies in 2% BSA, 2 mM CaCl_2_ in TBS for 1 h at room temperature. After being washed three times with TBS the cultures were mounted with aqueous mounting medium containing DAPI (vector).

#### *In vitro* whole brain

Young adult Hartley guinea pigs (150–300 g, Charles River) were used for IWB recordings. All procedures were approved by the Italian Department of Health and were conducted in accordance to FELASA guidelines and Italian and European directives (DL 116/92 and 2010/63/EU). Animals were anesthetized with sodium thiopental (125 mg/kg, i.p.) and transcardially perfused with a cold (4°C), oxygenated (95% O_2_, 5% CO_2_) saline solution composed of 126 mM NaCl, 3 mM KCl, 1.2 mM KH_2_PO_4_, 1.3 mM MgSO_4_, 2.4 mM CaCl_2_, 26 mM NaHCO_3_, 15 mM glucose, 2.1 mM HEPES, and 3% dextran (MW 70,000). The pH of the solution was corrected to 7.1 with 1N HCl. After assessing the absence of nociceptive and ocular reflexes, the brain was gently dissected out of the skull, transferred to a recording chamber, and perfused at 7 ml/min with the above solution (pH = 7.3, 15°C) via a peristaltic pump (Minipulse II, Gilson, France) through a cannula inserted in the basilar artery (Figure 5). Prior to recording, the temperature of the preparation was gradually increased to 32°C (0.2°C/min) (Llinas et al., 1981; Muhlethaler et al., 1993; De Curtis et al., 1998). In order to induce an ischemic insult in the hippocampal formation, a silk thread was positioned under the left rostral and caudal posterior cerebral arteries [r- and c-PCA, see Librizzi et al. (1999)] and a loose knot was prepared around the vessels. The flow was interrupted by pulling the thread ends to tighten the knot (Figure 5) (Pastori et al., 2007).

### Read-out systems

#### Optical manipulation and recording system for *in vitro* neural networks

The optical system combined a laser dissector with a microscope for simultaneous fluorescence and bright field imaging during electrophysiological recording of neural network activity, as previously described (Difato et al., 2011a).

The light source used to perform calcium fluorescence imaging was composed of TTL modulable laser diodes (TECBL-15 G-473-TTL-FC, World Star Tech. Inc., USA) coupled to the microscope (BX51, Olympus, Italy) through a circle top-hat engineered diffuser (ED1-C20-MD, Thorlabs, Optoprim, Italy) to remove laser speckles. A pair of UV doublets (Thorlabs, Optoprim, Italy) coupled the laser light to the microscope objective (60×, 0.9 NA water dipping). The laser light was focused on the back focal plane of the microscope objective to produce a homogenous wide field illumination on the sample. A light emitting diode at 590 nm wavelength served as the bright field illumination source (M590L2, Thorlabs, Optoprim, Italy). The wavelength of the diode was chosen to avoid interference with the emission spectra of the fluorochrome (Fluo4-AM, Invitrogen) used to label the sample. A dichroic mirror separated the light coming from the sample (green and red portion of light spectra) onto two cameras. Green emission light was deviated on CCD1 (V887ECSUVB EMCCD, Andor, Lot Oriel, Italy) acquiring the calcium fluctuations due to network activity, and the red portion of the light spectra was deviated on CCD2 (Pilot PIA1000-48GM, Basler, Advanced Technologies, Italy) to perform bright-field imaging. The CCDs image acquisitions and light sources were synchronized with a TTL signal coming from a D/A board (PCI-6529, National Instruments, Italy). The use of TTL-modulable light sources for fluorescence and bright field imaging allowed a precisely timed illumination of the sample, thereby reducing phototoxicity and facilitating long term calcium imaging of neural networks. Bright-field images were acquired at 1 Hz to detect network topography before and after laser dissection of network connections. Cells were previously incubated for 10 min with 5 μm Fluo-4 AM (Invitrogen, Italy). To monitor the neural network activity before and after laser induced network lesions, calcium imaging was performed at 60 Hz (light exposure of 3 ms each frame, at an average power at the sample of 60 μW).

Cells were kept under the microscope at 35°C using a Peltier device (QE1 resistive heating with TC-344B dual channel heater controller, Warner Instruments, Italy). For neuronal cultures plated on Petri dishes, pH and humidity were controlled by aerating a custom-designed polydimethylsiloxane (PDMS) sleeve, which integrated the objective for optical access, with humidified carbogen (95% O_2_, 5% CO_2_).

A pulsed, sub-nanosecond UV Nd:YAG laser at 355 nm (PowerChip nano-Pulse UV laser PNV-001525-040, Teem Photonics, Italy) served as the source for performing laser micro-dissection experiments. The diaphragm of the epi-illuminator was substituted by a narrow-band laser mirror, which reflects 355 nm laser light while passing all other wavelengths coming from the laser diodes used for fluorescence microscopy (DM6, TLM1-350-45-P, CVI, Italy), thus allowing fluorescence imaging and laser dissection to be performed simultaneously. Damage to neural network was inflicted with laser pulse repetition rate settled at 100 Hz, and an average power at the sample of about 4 μW.

#### Electrophysiological system for the *in vitro* whole brain

Extra- and intracellular recordings were performed simultaneously in piriform and medial entorhinal cortex (PC and m-ERC). To test the viability of the preparation throughout the experiment, we monitored evoked local field potentials (LFPs) in PC and m-ERC in response to the electrical stimulation (0.5–3 mA, 0.3 ms) of the lateral olfactory tract (LOT) using custom-made bipolar electrodes made of twisted, insulated silver wires. Intracellular recordings were performed with sharp micropipettes filled with 3M potassium acetate (input resistance 70–110 MΩ) and attached to an electronically controlled micromanipulator (Sutter Instruments, Novato, CA, USA). Signals were amplified by an intracellular amplifier (IR-283A Cygnus Technology, PA, USA). Field potentials were recorded using glass pipette filled with 0.9% NaCl (resistance 2–5 mΩ) or microwire arrays (Tucker-Davis Technologies, Alachua, FL, USA) featuring 16 tungsten planar recording wires (filament diameter 50 μm, tip angle 45°), each separated by 250 μm (impedance 30–40 KΩ). The extracellular signals were acquired using a PBX3 preamplifier (Plexon, Dallas, TX, USA) configured to separately process spikes (150 Hz–8 KHz bandwidth) and local field potentials (0.7–300 Hz).

Data were digitized at 25 kHz using a PCI-6071E A/D board (National Instruments, Austin, TX, USA) and stored on the hard drive of a personal computer. Recordings were performed using ELPHO software developed by Dr. Vadym Gnatkovsky at the C. Besta Neurological Institute (Milan, Italy).

### Computational model

In the following sections we will present the computational model used to mimic the dynamics expressed by finite size networks (cf. section “Experimental Models”).

#### Neuron model

The neuron model used for the finite size networks is based on the Izhikevich equations (Izhikevich, 2003). The dynamics of this model depend on four parameters that, correctly chosen, reproduce the spiking behavior and voltage traces of specific types of cortical neurons. From a mathematical point of view, the model is described by a two-dimensional system of ordinary differential equations.

(1) $$\frac{dv}{dt}=0.04v^{\text{2}}+5v+140-u+I_{\text{syn}}+I_{\text{noise}}$$

(2) $$\frac{du}{dt}=a(bv-u)$$

with the after-spike resetting conditions:

(3) $$\text{if}\;v\geq 40\text{mV}\to \{\begin{array}{l} \begin{array}{l} v\leftarrow c\\ u\leftarrow u+d \end{array} \end{array}$$

In Equations (1–3), *v* is the membrane potential of the neuron, *u* is a membrane recovery variable which takes into account the activation of K^+^ and inactivation of Na^+^ channels; *I*_syn_ describes the synaptic input from other neurons; *I*_noise_ is a current source generator introduced to model the spontaneous subthreshold electrophysiological activity of the neurons. Practically, we introduced a stochastic source of noise (modeled according to an Ornstein-Uhlenbeck process) to each neuron described as follows:

(4) $$dI_{\text{noise}}=-\frac{I_{\text{noise}}}{\tau_{I}}dt+\frac{m_{I}}{\tau_{I}}dt+s_{\text{1}}\sqrt{\frac{\text{2}dt}{\tau_{I}}}\xi_{t}$$

In Equation (4) the quantity ξ_*t*_ is a white noise with zero mean and unitary variance. In this way, *I*_noise_ is Gauss-distributed at any time *t* and, after a transient of magnitude τ_*I*_ (correlation length), converges to a process with a mean equals to *m*_*I*_ and standard deviation *s*_*I*_. For the simulation, we set τ_*I*_ = 1 ms, *m*_*I*_ = 25 pA, and *s*_*I*_ = 9 pA.

Among the possible firing patterns generated by the neuron model of Equations (1, 2), we implemented the family of regular spiking (RS) and the family of fast spiking neurons (FS) in percentage of 75% and 25%, respectively, in agreement with the experimental findings (cf. section “Finite Size Network Dynamics”). Mathematically, the four aforementioned parameters were set as follows:

(5) $$\begin{array}{ll} a=\left[\begin{array}{l} 0.02\\ 0.02+0.08r_{i} \end{array}\right]\; & \;b=\left[\begin{array}{l} 0.2\\ 0.25-0.05r_{i} \end{array}\right]\\ c=\left[\begin{array}{l} -65+15r_{i}^{2}\\ -65 \end{array}\right]\; & d=\left[\begin{array}{l} 8-6r_{i}^{2}\\ 2 \end{array}\right] \end{array}$$

In Equation (5), the first row is relative to the excitatory, while the second one to the inhibitory neurons. *r*_*i*_ is a random variable which spans from 0 to 1, and *i* the neuron index. *r*_*i*_ was added in order to introduce a further variability in the neuron dynamics: for example, a neuron exhibits classic RS behavior if *r*_*i*_ = 0, and bursting behavior if *r*_*i*_ = 1.

#### Finite size network model

Graph theory was used to represent the network connectivity. All graphs are defined by nodes which represent the neurons, and edges which model the morphological connections among the neurons. The structure of the graph is described by the adjacency matrix, a square matrix of size equal to the number of nodes *N* with binary entries. If the element *a*_*ij*_ = 1, a connection between the node *j* to *i* is present, otherwise *a*_*ij*_ = 0 means no connection. All the auto-connections are avoided (*a*_*ii*_ = 0, ∀ i). Then, the value 1 of the non-zero *a*_*ij*_ elements has been replaced to mimic different synaptic strengths. Synaptic weights were chosen randomly from a normal distribution with a mean value and standard deviation equal to 10 and 3.5, respectively.

To model the synaptic transmission we chose the approach of the pulse-coupled neural networks: practically, the firing of the *j*-th neuron causes an instantaneous change in the membrane potential of the neuron *i*-th by means of the weight *s*_*ij*_.

Among the possible graphs, following the experimental findings regarding the functional connectivity of such confined neuronal assemblies (cf. section “Finite Size Network Dynamics”), we implemented neuronal networks with a scale-free (SF) connectivity (Barabasi and Albert, 1999). Briefly, in SF networks the degree distribution follows a power law: if *m* is the number of edges incident to a node, i.e., the degree, the power law distribution is given by Dorogovtsev and Mendes (2002):

(6) $$P(m)=m^{-\gamma }$$

where γ is the characteristic exponent. This law suggests that most nodes have just a few connections and other, named *hubs*, have a very high number of links. To build a SF network, we made use of the algorithm described in Batagelj and Brandes (2005), particularly efficient in terms of computation when dealing with large-scale networks. Nodes are added successively. For each node, *m* edges are generated. The endpoints are selected from the nodes whose edges have already been created, with a bias toward high degree nodes.

In order to mimic the experimental conditions of the confined assemblies described in section “Finite Size Network Dynamics,” in section “Simulation Results” we presented the results regarding the ongoing activity of networks made up of 90, 100, 120, 150, 240, 320, and 520 neurons.

### Data analysis and statistics

#### Analysis of network dynamics based on calcium fluorescence imaging

Custom software running in MATLAB (Crépel et al., 2007; Bonifazi et al., 2009) was used for the automatic identification of the cells loaded with the calcium indicator and for the extraction of their fluorescence signals as a function of time (time resolution 59 Hz). To detect the calcium events (i.e., the onset and offset of neuronal firing) from the fluorescent trace *F*_*ij*_ of the neurons (1 ≤ *i* ≤ *M, M* number of neurons; 1 ≤ *j* ≤ *N, N* number of frames) we calculated the first derivative of the fluorescent signal (ΔF_*ij*_ = *F*_*ij*+1_ − *F*_*ij*_) and we integrated Δ*F*_*ij*_ in overlapping sliding time windows of 1 s (*I*_*ij*′_ = Σ_*j*′ ≤ *n* ≤ *j*′ + 59_ Δ*F*_*nj*_; 1 ≤ *j*′ ≤ *N* − 59). A Gaussian fit centered at zero was used to extract the standard deviation σ_*i*_ of the noise of the processed signal *I*_*ij*_. Signal transients exceeding the threshold of 3σ_*i*_ for at least 5 consecutive points were considered as calcium events. The onset and the offset of these calcium events were determined using a four-parameter sigmoidal equation as described in Takano et al. (2012). The estimated onset and offset times were fixed respectively to the 5% and the 95% of the sigmoidal plateau.

The reconstruction of the functional connectivity of the network was based on pair-wise correlation analysis of the onset time series extracted from the calcium imaging data, as described in Bonifazi et al. (2009). Briefly, when the firing onset of cell *j* preceded in a repetitive way the firing onset of cell *k*, a functional connection directed from *j* to *k* was established. In order to reveal these temporal correlations, the post-stimulus time histogram of cell *k* centered on the firing onsets of cell *j* was calculated within a maximal time lag of 500 ms. Both the Student's *t*-test and the Kolmogorov-Smirnov test with a level of confidence of 5% were used to exclude the possibility that the poststimulus time distribution could be a Gaussian distribution with zero mean or a uniform distribution, respectively. In this way, we excluded cases where the activation of two neurons was completely uncorrelated (uniform distribution) or synchronous (Gaussian centered at zero).

The cross correlation between firing onsets time series of individual neurons was used to estimate the average correlation and average time of activation of each neuron relative to all others, similarly to what described in Bonifazi et al. (2009) and Marissal et al. (2012). Briefly, the cross correlation between the time series of neurons *a* and *b* was calculated as follows:

(7) $$CC_{ab}(\tau )=\frac{\sum_{0<t<T}\left(a_{t\;+\;\tau }-<\text{​}a\text{​}>\right)\cdot (b_{t}\;-<\text{​}b\text{​}>)}{\sigma_{a}\cdot \sigma_{b}}$$

where σ_*a*_ and σ_*b*_ are the standard deviation of the time series, *t* is the sampling time, *T* the duration of the entire movie and |τ| ≤ 1 s.

The maximum cross-correlation value (*CC*^max^_*ab*_) and the time lag of its occurrence (τ^max^_*ab*_) were used to calculate, respectively, the average correlation and average time of activation of neuron *i* to the following formulas $\langle CC_{i}^{\max}\rangle =\frac{1}{n}\sum_{j\neq i}CC_{ij}^{\max}$ and $\langle \tau_{i}^{\max}\rangle =\frac{1}{n}\sum_{j\neq i}\tau_{ij}^{\max}$ where *n* is the number of neurons displaying a positive cross-correlation with neuron *I*.

#### Processing of electrophysiological signals from the IWB

Raw data acquired by the ELPHO software were loaded into MATLAB (Mathworks Inc., Natick, MA, USA) for off-line processing. First, raw traces were band-pass filtered to select either multi-unit activity (MUA, 800 Hz–3 KHz) or local field potentials (LFP, 1–300 Hz). Stimulation artifacts were suppressed using an off-line MATLAB implementation of the SALPA algorithm (Wagenaar and Potter, 2002). Highly noisy channels were visually excluded from the analysis. Then, MUA raw data were spike-detected by means of the PTSD algorithm (Maccione et al., 2009) (peak lifetime period = 2 ms; refractory period = 1 ms; threshold = ±8 times the estimated noise standard deviation). The result of the spike-detection procedure consists of a series of point processes (i.e., spike trains), one for each recording channel (Bologna et al., 2010).

We evaluated the network-wide evoked response by computing the Peri Stimulus Time Histogram (PSTH; Perkel et al., 1967) for each recording channel of the array and for the full array [time bin = 4 ms, time window = (−100 ms, +400 ms) relative to the stimulus onset]. We also measured the intensity of the response as the average number of evoked spikes in a 200-ms time window following each stimulus. The final dataset comprised 4 recordings in control brains (duration ~300 s, 10–20 paired pulses delivered to the LOT at 0.05 Hz, inter-pulse interval 200 ms) and 3 recordings before and after the induction of focal ischemia (same stimulation protocol).

## Results

### Finite size network dynamics

#### Spontaneous synchronizations in finite size networks

**Figure 2:**
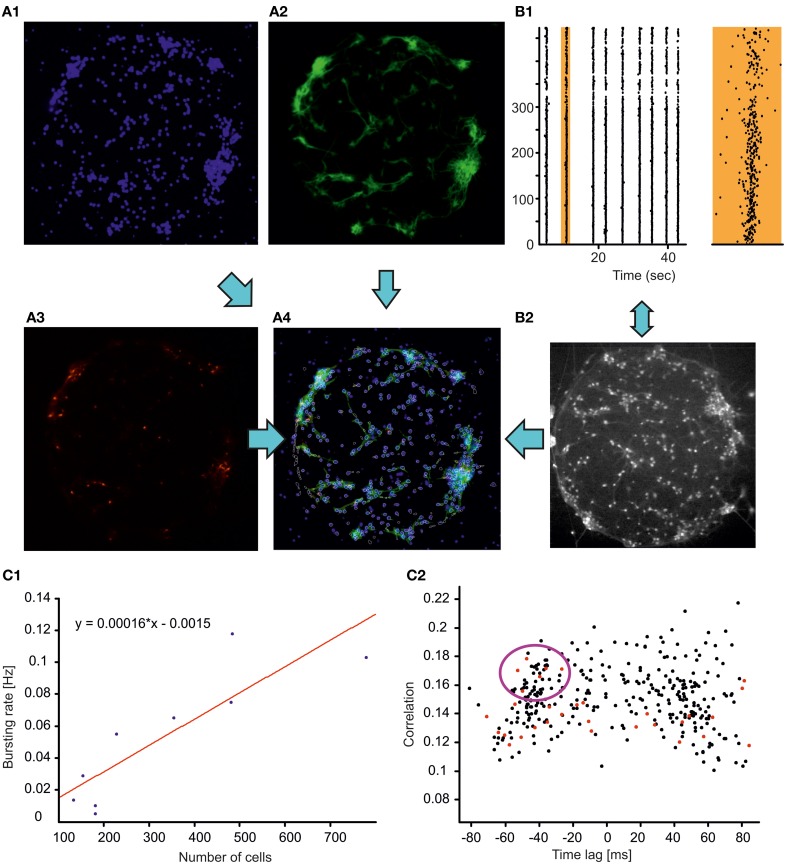
**Structure *vs*. function relations in neocortical finite circuits. (A)** Immunocytochemical staining revealing cellular nuclei (blue, DAPI, **A1**), neuronal cells (green, MAPs, **A2**), GABAergic neurons (red, GAD67, **A3**). In panel **(A4)**, the contours of the cells monitored through calcium imaging (white) are superimposed to the merged immunocytochemical pictures. **(B)** Monitoring the dynamics of the neuronal circuit through calcium imaging. Raster plot (**B1** left plot) of the activity of the circuit (shown in panel **A**) displaying stereotyped spontaneous network synchronizations (broken vertical lines). The activity of a representative network synchronization (marked in orange) is shown with higher temporal resolution on the right orange plot (bottom scale bar 0.5 s). The cells loaded with the calcium indicator OGB are shown in the panel **(B2)** (objective magnification 10×, field of view 800 × 800 μm). **(C1)** Frequency of spontaneous synchronizations as a function of circuits' population size (blue dots, *n* = 9). The cell number was estimated by counting the cellular nuclei stained with DAPI. The result of the linear fit with least-squares regression (Pearson correlation coefficient 0.88) is represented by the red line and by the equation. **(C2)** Time lag—correlation graph for the circuit shown in **(A)** plotting for each imaged neuron the average correlation and average time of activation relative to all other cells (see section “Materials and Methods”). Red dots indicate GABAergic cells. The violet circle highlight GABAergic cells reliably activated at the synchronization build up possibly playing a key role in the orchestration of network synchrony similarly to what previously documented for the developing hippocampal circuits (Bonifazi et al., 2009).

To build an experimental model for the study of physiological and impaired communications between neuronal assemblies we grew finite size neuronal networks, i.e., networks composed of neuronal assemblies spatially separated by hundreds of micrometers and interconnected through long neuritis. As a first step, we focused on the properties of single modules, i.e., the structural and dynamical properties of isolated and spatially confined neuronal circuits (Figure 2). Isolated neuronal circuits located within an 800 × 800 μm spot were obtained by plating the cells on glass cover slips previously coated with a geometrically defined molecular adhesive layer (PDL). The individual cell populations varied between a few dozen up to a few hundred neurons. Similar to homogenous and clustered cultures (Chiappalone et al., 2006; Shein-Idelson et al., 2010), finite size circuits displayed spontaneous synchronized events after 2 weeks in culture (Figure 2, panel B1) occurring with a frequency linearly correlated with the number of cells present in the circuit (Pearson correlation 0.88, Figure 2C1). Likewise, depending on the density of the plating and on the vicinity to the supporting network, finite size circuits organized into monolayers or in three-dimensional clusters, with a higher propensity of clustering at increased plating density or at larger distances from the supporting network (data not shown). We used calcium imaging of monolayer neuronal circuits (performed with a 10× objective) in combination with immunocytochemical staining to map the functional and structural properties of all the neurons in the circuits with single-cell resolution. GABAergic cells could be specifically identified (Figure 2A3), allowing us to investigate their specific involvement in spontaneous synchronization processes, similar to the work of Bonifazi et al. (2009) in developing hippocampal networks.

A pair-wise analysis based on the cross-correlation between the firing onsets time series of pairs of neurons (see section “Materials and Methods”) was used to estimate the average correlation and average time of activation of each neuron relative to all others (Bonifazi et al., 2009; Marissal et al., 2012). In all the circuits analyzed (*n* = 4) the time correlation graph presented a bimodal distribution (Figure 2C2), indicating that network events synchronized first the population of neurons plotted on the left side of the graph (i.e., with a time lag < 0), whereas neurons on the right (i.e., with a time lag > 0) were activated next. In addition, the presence of highly correlated early activated GABAergic neurons was observed (red points within the violet circle in Figure 2C2). Interestingly, the existence of a characteristic, early-activated neuronal population within the network synchronizations has been already documented in developing hippocampal circuits (Bonifazi et al., 2009) even in absence of GABAergic transmission (Marissal et al., 2012). Notably, in the presence of GABAergic transmission it has been shown that early-activated GABAergic neurons can play the role of hub cells in orchestrating network dynamics (Bonifazi et al., 2009). The similarity between these previous observations and the results presented here suggest that cortical circuits share common innate features in their functional organization.

#### Effect of laser ablation on functional connectivity

To monitor the synaptic re-organization of lesioned neuronal circuits with single cell resolution, we reconstructed the functional connectivity of a neuronal subset of a larger neuronal network 20 min before and after laser-induced ablations (see section “Materials and Methods”).

**Figure 3:**
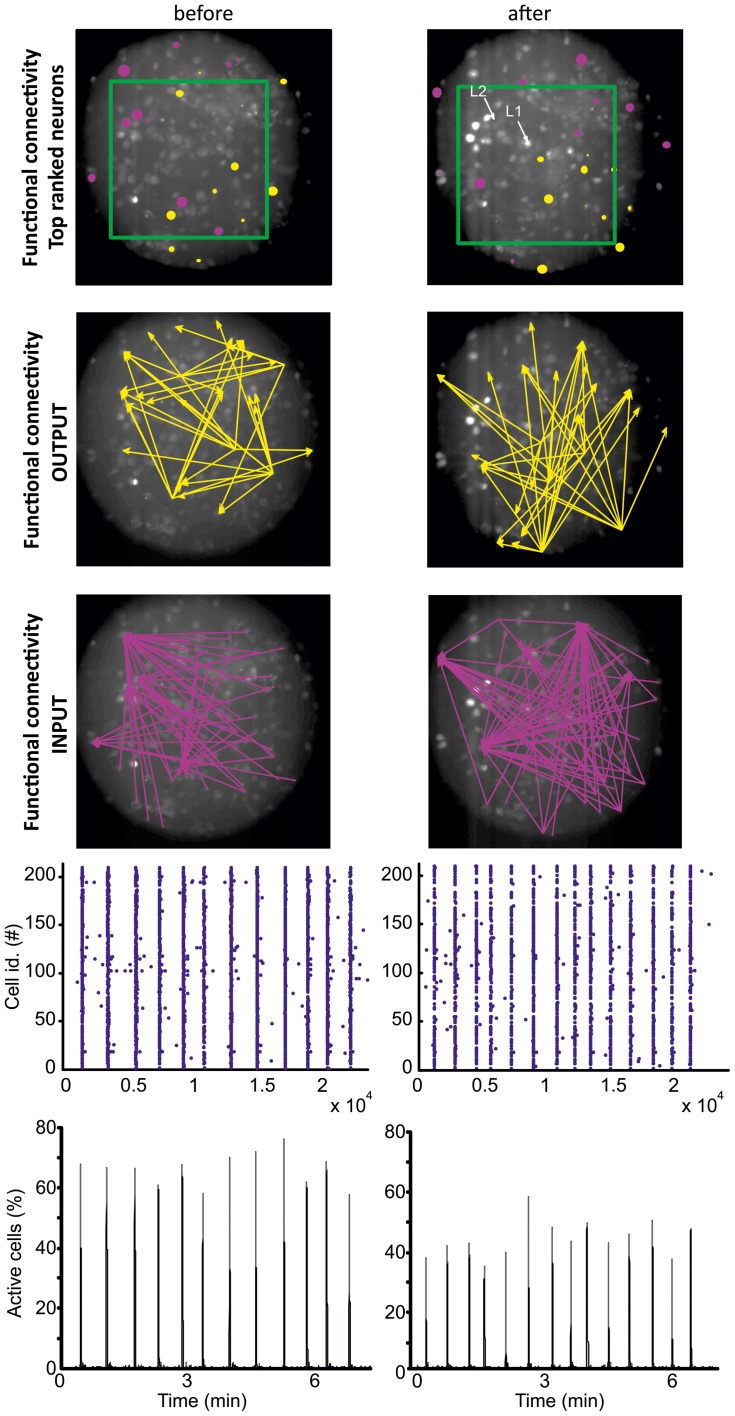
**Directed functional connectivity before (left) and after (right) lesion.** The number of OUTPUT and INPUT functional connections has been calculated for all the imaged neurons based on the temporal correlation between the firing onsets of the neurons (see section “Materials and Methods”). The ten top ranked cells, i.e., the cells with the largest number of functional OUTPUT (yellow) and INPUT connections (pink), are represented in the top row. For graphic clarity, the connectivity graphs shown in the 2nd and 3rd rows (respectively INPUT and OUTPUT connections) include only the five top ranked cells. The data refers to a homogenous neuronal network where functional hub cells (i.e., neurons with a very large number of functional connections) were not identified. The fluorescent images show the cells loaded with the calcium indicator Fluo4 (see section “Materials and Methods”). The locations of the two lesions (L1 and L2) are marked by the white arrows. The green rectangle highlights the region shown in Figure A1. The field of view is a circular region of 244 μm diameter. The raster plot (representing the firing onsets) and the fraction of activated cells are shown respectively in the 4th and 5th row.

**Figure A1:**
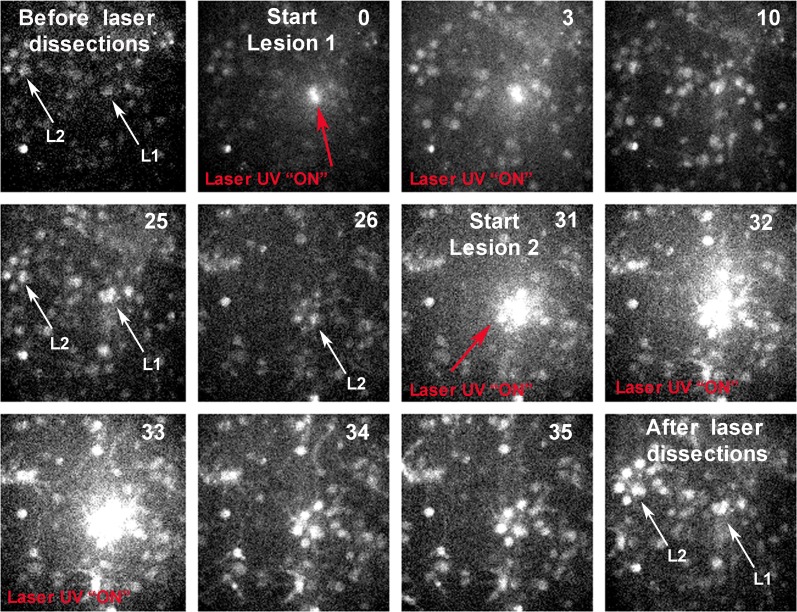
**Subpopulation of a neuronal network before and after laser induced lesions.** The first panel, starting from the left upper corner, shows a subpopulation of a neuronal network loaded with Fuo4-AM. White arrows, depicted with L1 and L2, indicate the positions of the lesions inflicted to the network. The red arrows indicate the position of the UV laser focus spot. The average power delivered at the sample, during lesion one, is 4 μW, and during Lesion 2, is 5 μW. We delivered 300 UV light pulse for each lesion, at pulse-repetition rate of 100 Hz. At 25 s, after Lesion 1, the L2 position is centered onto the UV focus spot. The last panel shows the same field of view of the first one, after laser inflicted damages. The cells directly affected by the UV laser presented saturated calcium signal. Numbers indicate seconds. The field of view is 150 × 150 μm. Calcium imaging was acquired at 60 Hz.

Two micro-lesions (lesion 1 and lesion 2) were induced next to the center of the field of view, using an average laser power at the sample of 4 μW and 5 μW, respectively. The second lesion was performed at higher power to obtain a more pronounced alteration of the network. Indeed, this lesion produced a strong intracellular calcium increase in several cells, and a calcium “shockwave” started to propagate through the network. After a few minutes, only directly ablated cells displayed a saturated calcium fluorescence signal, while the other neurons recovered a relatively low basal calcium level and presented spontaneous activity (cf. Figure A1). The frequency of occurrence of spontaneous network synchronizations was not affected by the lesions (Figure 3, 4th and 5th rows) with no significantly statistical difference between the inter-burst interval distribution before and after lesion (student *t*-test, *p* > 0.05). However, the number of cells recruited within the network events in the imaged field (i.e., close to the location of the lesion) decreased by 31 ± 10% (student *t*-test, *p* < 0.05).

Based on the calcium dynamics of the cells imaged in a circular field of 244 μm diameter (Figure 3), we reconstructed the functional connectivity of the neuronal population through a pair-wise analysis of the onset of firing (see section “Materials and Methods”). Briefly, if the activation of cell *i* reliably preceded the activation of cell *j* (i.e., over several repetitions with statistical significance, see section “Materials and Methods”), we inferred a functional connection directed from *i* to *j*. Cell pairs that were synchronously activated or not displaying any activation order were not included in the directed functional connectivity reconstruction (see section “Materials and Methods”). Figure 3 (1st row) shows the location of ten neurons with the highest number of functional INPUT (violet) and OUTPUT (yellow) connections before and after the lesions. Interestingly, after the lesions, top rank INPUT and OUTPUT neurons segregated into spatially distinct regions. Top rank OUTPUT neurons relocated in the bottom right region while top rank INPUT neurons remained in the rest of the circuit. In addition, just one out of the ten neurons for each group belonged to the top rank group before and after the lesion. The relocation of the functional connections (drawn for clarity just for the five best ranked neurons) can additionally be observed in Figure 3 (2nd and 3rd row).

### *In vitro* whole brain

**Figure 4:**
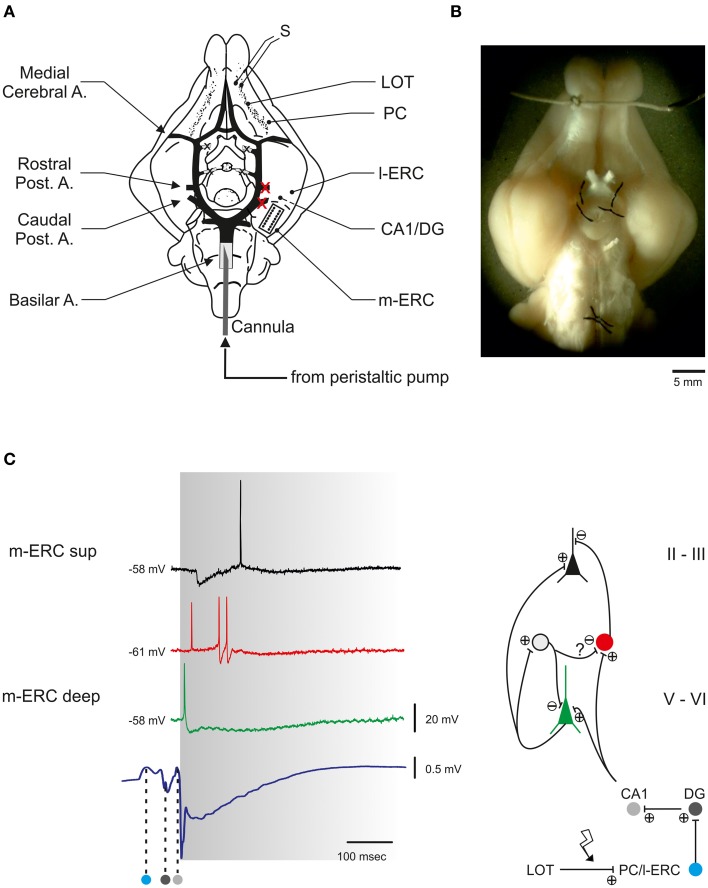
**The guinea pig isolated whole brain (IWB). (A)** Schematic view of IWB observed from its ventral surface. The circle of Willis with its principal branching arteries is highlighted in black. The whole brain is perfused by means of a peristaltic pump that delivers ACSF to the brain through a polyethylene cannula inserted into the basilar artery. The two vessels that are occluded to induce the hippocampal ischemia are marked by red crosses. In the same hemisphere a microelectrode array (MEA) is positioned in the center of the m-ERC (delimited by dotted line). S, stimulating electrode; LOT, lateral olfactory tract; PC, piriform cortex; l-ERC, lateral entorhinal cortex; DG, dentate gyrus; m-ERC, medial entorhinal cortex. **(B)** Stereomicroscope photograph of the isolated brain positioned in the perfusion chamber. **(C)** Electrical responses to LOT stimulation recorded in the m-ERC. Left, intracellularly recorded voltage traces from a superficial pyramidal cell lying at 200–300 μm from pial surface (black trace), a GABAergic interneuron (400–500 μm, red trace), and a deep pyramidal cell (600–1000 μm, green trace). Note the correspondence between the early firing of an action potential in the interneuron and an IPSP (asterisk) recorded in the superficial pyramidal cell. The bottom trace is an extracellularly recorded field potential (LFP) characterized by a volume conducted component propagating from the rostral part of the LOT-activated synaptic pathway (PC and l-ERC) and subsequently invading the hippocampal structure (DG and CA1, dark and light gray spots, respectively). The left margin of the gray area is aligned to the first component of the m-ERC LFP. Right, simplified scheme of the polysynaptic neuronal circuitry within the m-ERC, based on the evoked response pattern and delay analysis of the neuronal response to LOT stimulation. The gray cell represents a putative interneuron mediating a feedback GABAergic inhibition onto a deep pyramidal cell and a feed-forward inhibition onto another interneuron.

We also characterized the activity of an *ex vivo* experimental model (i.e., the isolated brain of a guinea pig, Figure 4) before and after a lesion induced by a focal ischemia.

#### Network response to LOT stimulation in the m-ERC

Electrical stimulation of the LOT induced a polysynaptic response in the m-ERC mediated by the interposed activation of the hippocampus (Biella and De Curtis, 2000; Gnatkovsky and De Curtis, 2006) (Figure 4). The intracellular correlate of the LOT-evoked delayed response in neurons of m-ERC superficial layers was characterized by an early GABA_A_ receptor- mediated inhibitory postsynaptic potential (IPSP; latency from LOT stimulation: 51 ± 1 ms, *n* = 12), followed by a relatively slow (duration 409 ± 36 ms) NMDA-dependent depolarizing component which often reached threshold for spike firing. Conversely, pyramidal cells in deeper layers responded to LOT stimulation with an early excitatory postsynaptic potential (EPSP) occurring 15 ± 1 ms after the population spike recorded in the dentate gyrus (DG, Figure 4). The EPSP often crossed the threshold for action potential firing and was followed by a relatively slow inhibitory potential mediated by GABA_B_ receptors (Gnatkovsky and De Curtis, 2006). The early inhibition of the superficial principal cells is presumably due to a feed-forward mechanism sustained by interneurons recorded in layers II/III (i.e., basket and chandelier cells; Canto et al., 2008). In Figure 4 the firing of an interneuron corresponds to the early IPSP measured in the pyramidal cells in the same layer.

**Figure 5:**
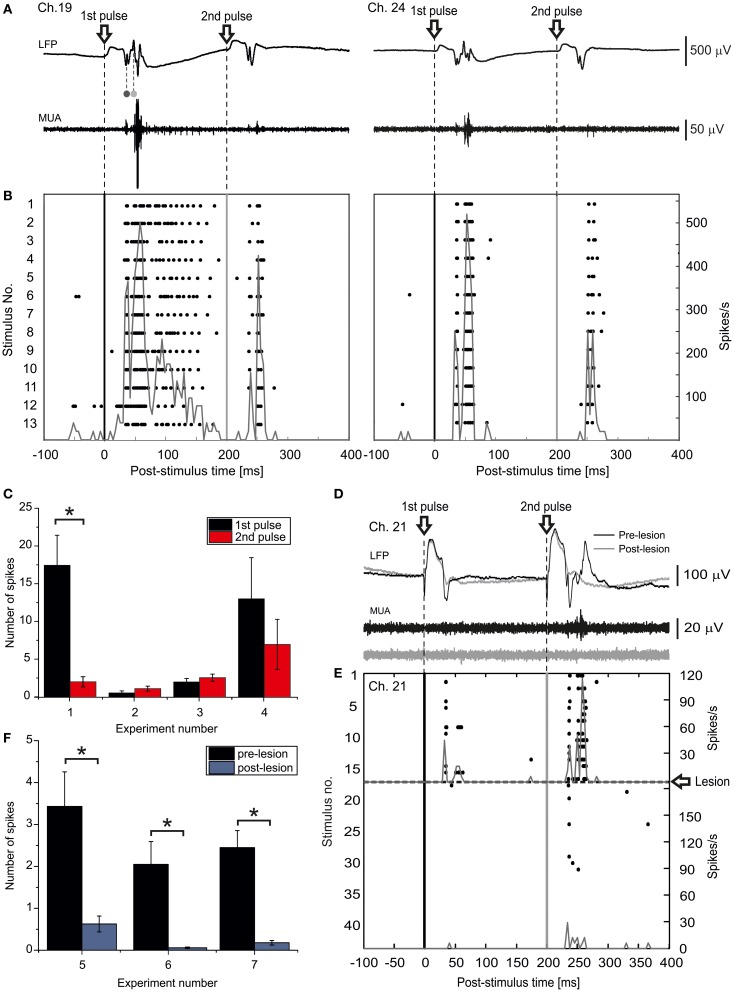
**LOT-evoked m-ERC network activity is abolished after ischemic lesioning of the hippocampus. (A)** Local field potentials (LFP) and multi-unit activity (MUA) raw traces from two selected electrodes (19 and 24, experiment #1) recorded in response to an individual paired-pulse stimulus (ISI 200 ms) delivered to the LOT. The volume-conducted components originating in DG and CA1 are indicated by the dark gray and light gray dots, respectively. **(B)** Peri-stimulus raster plots for the same two representative electrodes. The corresponding PSTHs are superimposed (bin size = 4 ms). **(C)** Summary plot of mean number of evoked spikes (mean ± S.E.M.) after 1st and 2nd pulse for all four experiments. ^*^p < 0.05, Mann–Whitney *U*-test. **(D)** LFP and MUA raw traces of one selected electrode recorded in response to a paired-pulse stimulus either before (black trace) or after (gray trace) an ischemic lesion of the hippocampus. **(E)** Peri-stimulus raster plot for the same representative electrode, before and after the lesion. The corresponding PSTHs are superimposed (bin size = 4 ms). **(F)** Summary plot of mean number of evoked spikes by a paired pulse stimulus delivered to the LOT (mean ± S.E.M.) either before or after the ischemic lesion of the hippocampus for all analyzed experiments. ^*^p < 0.05, Mann–Whitney *U*-test.

Spiking responses to paired-pulse LOT stimulation (inter-pulse interval 200 ms) were recorded by 16-channel MEAs implanted in the superficial layers of the m-ERC (200–500 μm from pial surface; Figure 5A). Figure 5B shows the peri-stimulus raster plots of two selected channels (19 and 24, experiment #1) in response to each of the two LOT stimulations for a selected experiment. An earlier phase, which we observed in almost all active recording channels, was characterized by two relatively sharp peaks: the first corresponding to the far-field response originating in the hippocampus and the other corresponding to the initial phase of the m-ERC response (Figure 4). This was followed by a late, long-lasting but less reliable component (cf. channels 19 and 24). The histogram in Figure 5C displays the number of spikes (mean ± S.E.M.) evoked by the 1st and the 2nd pulse for all experiments (control condition) as a measure of response intensity. In 2 out of 4 experiments (#1, #4) we observed a stronger activation after the 1st rather than 2nd pulse, whereas in the other 2 experiments (#2, #3) responses to the 2nd pulse were slightly stronger than to the 1st pulse (no significant statistical difference). However, one must consider that first evoked responses in experiments #1 and #4 were on average more intense, probably reflecting a relatively high probability of excitatory neurotransmitter release upon the first pulse. This would nearly deplete the available pool of synaptic glutamatergic vesicles, leading to a paired-pulse depression of the postsynaptic response.

#### Cutting the olfactory pathway: hippocampal focal ischemia

Occlusion of the posterior left cerebral arteries abruptly reduced ACSF perfusion of the hippocampus, resulting in a block of the propagation of the synaptic activity toward the entorhinal cortex (Figure 4). About 5 min after the ischemic insult, LOT stimulation failed to evoke any response (Figure 5). Stimulus-triggered raster plots and the corresponding pre- and post-lesion PSTH are shown in Figure 5E. The bar graph in Figure 5F summarizes the total number of spikes evoked by a paired-pulse stimulus before and after the ischemic lesion. A significant reduction of the response intensity caused by the lesion was observed in all three analyzed experiments.

### Simulation results

In this section, we report the results of simulations in which we modeled the effects changing the number of neurons in confined networks. Each simulation lasted 10 min, sampled at 10 kHz. Networks were simulated in MATLAB (The Mathworks, Natik, US). Peak trains were stored and then processed by using SpyCode software (Bologna et al., 2010), conveniently adapted to manage large-scale networks.

#### Dynamics of finite size networks

We simulated the ongoing activity of neuronal networks made up a 90, 100, 120, 150, 240, 320, and 520 neurons. The choice of these networks sizes followed from the experimental findings described in section “Finite Size Network Dynamics” (assuming a neuron/glia ratio equal to 2:1). In addition, 25% of such neurons were considered inhibitory (Isaacson and Scanziani, 2011) and were modeled as FS neurons (cf. section “Computational Model”).

**Figure 6:**
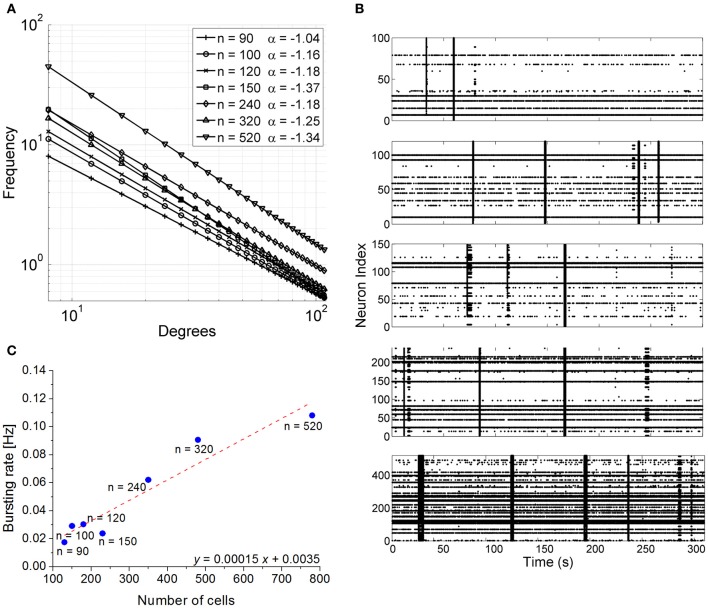
**Simulation results. (A)** Degree distribution of the 7 scale-free networks. **(B)** Raster plots showing 300 s of spontaneous activity of simulated confined networks. Each raster is relative to a different network size. From top to bottom networks with 100, 120, 150, 240, 520 neurons can be observed.. IFR profiles was evaluated over simulations lasting 600 s (bin size = 100 ms). **(C)** Bursting rate frequency as a function of network population size. The number of neurons of the simulated networks was reported near the blue dots. The *x*-axis reports the total number of cells (glia + neurons) in order to make easier a comparison with Figure 2C1. The result of the linear fit with least-squares regression (Pearson correlation coefficient 0.96) is represented by the red-dotted line and by the fitting equation.

Model neurons were connected following a scale-free (SF) topology. Figure 6A shows the degree distribution of the simulated SF networks. For all SF networks, the degree distribution was fitted by a power law and the corresponding exponent lay between −1.04 (network made up of 90 neurons) and −1.34 (networks made up of 520 neurons).

The simulated networks displayed spontaneous synchronized events (network bursts) independently of their size (Figure 6B). However, the frequency of occurrence of those synchronized events varied in a linear manner with respect to the number of cells present in the circuit (Pearson correlation 0.96, Figure 6C). To facilitate comparison with Figure 2C1, the *x*-axis of Figure 6C reports the total cell number (neurons + glia), although the number of neurons effectively simulated is indicated near the blue dots. The results of the simulation were fit well with the experimental data, as confirmed by the slope of the linear fit (0.00015 vs. 0.00016). An interesting finding was that the simulated networks tended to show a higher proportion of random spiking activity and less bursting than normally observed in actual finite-size neuronal networks. This is consistent with other experimental results of interconnected finite-size networks previously reported in the literature (Macis et al., 2007).

### Hardware set-up for a brain prosthesis

The hardware set-up that will be used to interface the biological component (either the neuronal culture or the *in vitro* whole brain) is a Spiking Neural Network (SNN) system. This SNN implements biologically realistic neural network models, spanning from the electrophysiological properties of one single neuron up to network plasticity rules. As already discussed in the modeling section, the choice of Izhikevich neuron model is relevant because (1) it is biologically realistic, and (2) it operates in biological real time. By real-time, we mean that computation results are provided within a firmly controlled delay (10 ns precision), which is lower than the sampling period (100 μs to 1 ms). Among these modules, the computation-critical task is the implementation of a SNN model, which represents the prosthesis itself, and the analysis of biological signals to produce events from the recorded activity.

**Figure 7:**
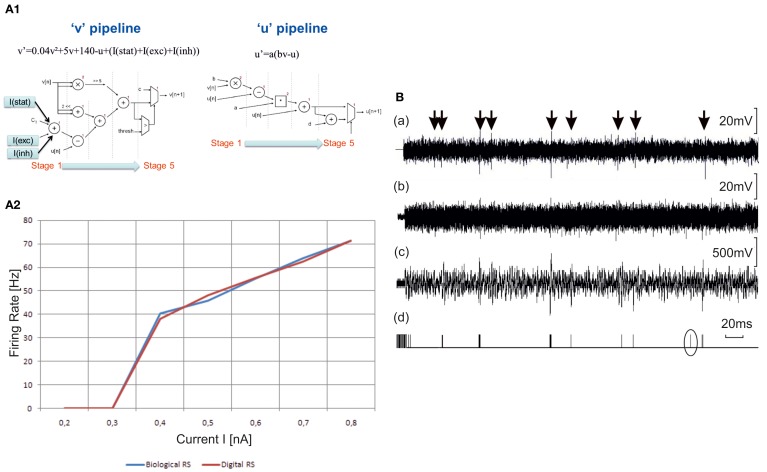
**Hardware elements for the neuro-prosthesis. (A1)** Choice of the topology. To implement the two equations of Izhikevich model, two topology of pipeline are chosen (Cassidy and Andreou, 2008). There are five stages of computing for each equation. The I(stat), I(exc), and I(inh) currents describe the synaptic contribution. **(A2)** Hw-based model. Comparison of f(I) curves between biological Regular Spiking (RS) neuron and digital one. The biological curves are intracellular recordings of regular-spiking neurons in ferret visual cortex *in vitro*. The neuromorphic board gives the same results in term of frequency of the neuron *vs*. the stimulation current. **(B)** Outputs of the detection system to be implemented in the closed-loop set-up of the brain prosthesis. *First row*—*(a)*. Raw electrophysiological signal. *Second row—(b)*. The same signal with added Gaussian white noise to reduce Signal to Noise Ratio. This step was added to stress the capability of the system to detect action potentials in difficult conditions. *Third row—(c)*. Output of the stationary wavelet decomposition preprocessing module. We used a Haar mother wavelet with 16 bits fixed point computation. The output signal is the sixth level detail output of the decomposition tree. *Fourth row—(d)*. Binary output of the detection module. This output is the result of a threshold applied to the signal in (c). The threshold is computed from the standard deviation of the first level detail output of the wavelet decomposition tree. The emphasized detected spike is a false positive. This shows that the signal *(b)* represents the limit of signals that can be reliably processed by our system. These signals were first recorded then input to the system with a waveform generator.

The digital Izhikevich neurons and detection system are implemented as a configurable digital integrated circuit (field-programmable gate array, FPGA) using the VHDL language. We implement Regular Spiking (RS) neurons (excitatory) and Fast Spiking (FS) interneurons (inhibitory) similar to those found in cell culture (Figures 2, 3 and 6). The hardware models follow the Izhikevich equations with parameters corresponding to RS activity (*a* = 0.02, *b* = 0.2, *c* = −65, and *d* = 8). In Figure 7A1 we describe the choice of the topology (Cassidy and Andreou, 2008) to implement the Izhikevich equations. We implement a neuron on FPGA board Xilinx Virtex 5 XC5VLX50. This neuron uses really few resources (only 2% of the FPGA) and works in real-time. In Figure 7A2 we compare the behavior f(I) of biological RS neurons and one RS neuron implemented into the FPGA.

Concerning the SNN, our goal was to implement a model using 80 neurons (FS and RS) with high connectivity capacity (e.g., 6400 synapses). Network structure is fully configurable, and synapses are excitatory or inhibitory conductances which provide current depending on the postsynaptic membrane voltage. Delays are also implemented to provide good accuracy on timing. The network is defined into the RAM of the digital board where lie all characteristics of all neurons and synaptic connections in the network. A synaptic connection is defined by a synaptic weight and the address of the neuron linked by this synapse. Added with complementary functions like loopback stimulation and monitoring, this system will be able to perform cross-platform neural computation.

The detection of neural electrophysiological activity is done by a reconfigurable acquisition based on wavelet detection circuit for *in vitro* biological signals. Our strategy for real-time spike detection is to implement a pre-processor, which emphasizes spikes shapes and attenuates out-of-band noise. This pre-processor provides two outputs corresponding to different wavelet detail levels. The first one is essentially composed of out-of-band noise used to determine a threshold level adapted to the signal amplitude. The second output is compared to the threshold to discriminate spike events. The pre-processing algorithm is the Stationary Wavelet Transform (SWT). The detection system computes in real-time the SWT, the adaptive threshold and the comparison. This method proved to be very efficient to extract action potential of excitable cells from very noisy signals (Raoux et al., 2012). Figure 7B shows the performance of the method on a single channel setup. Action potentials are emphasized by arrows on the signal A. We added significant noise [signal (b)] and then sent the signal to the detector that provides outputs (c) and (d).

To summarize, all modules (i.e., Izhikevich neuron, neural network specifications, detection and stimulation modules) will be implemented into the FPGA. This modular system will be used as a cross-platform neural computation unit. Microelectrode arrays will be used to record and electrically stimulate living neural networks, with a specific emphasis on stimulation localization. Dedicated integrated electronics will be designed to implement the communication channels between the living and the artificial networks. The biological signals (from living to artificial) will be processed by using on-line spike detection algorithms and a rate-based decoding (Rieke et al., 1997; Novellino et al., 2007; Tessadori et al., 2012), while the firing rate of an artificial neuronal sub-network will be translated into the stimulation frequency for the biological network (from artificial to living), thus following a similar rate-based strategy. The system including the artificial and living neural networks will form a closed loop with a regulated feedback (cf. next Section).

### A bi-directional neuro-prosthesis

**Figure 8:**
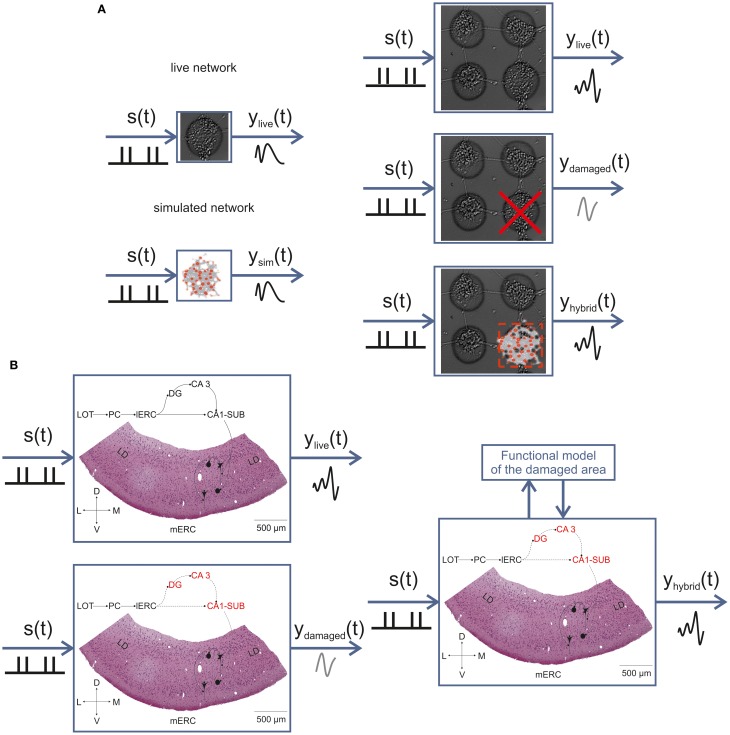
***In vitro* neuro-prostheses.** Sketch illustrating the main approach of the BRAIN BOW project. First, we will characterize the I/O function of simple finite-size networks and reproduce it by means of a computational model (**A**, left). Second, we will use more complex modular networks and replace one sub-network module with our computational model of the finite-size network, in order to replicate the function of the intact system (**A**, right). Finally, the same conceptual approach will be adopted to recover the function of the olfactory-limbic circuit after an ischemic lesion of the hippocampus **(B)**. The bidirectional interaction with a model reproducing the function of the damaged area will allow restoring the original I/O pattern. s(*t*): stimulus function; *y*_live_(*t*): response function of a healthy preparation; *y*_sim_(*t*): response function of the neuronal network model; *y*_damaged_(*t*): response function after lesion in the IWB; *y*_hybrid_(*t*): response function of the hybrid system resulting from the combination of biological and artificial components. In panel **(B)**, the hippocampal areas targeted by the ischemic lesion are marked in red.

The knowledge that we gained through the various studies presented here will contribute to the final realization of a bi-directional communication between *in vitro* and *in silico* models of interconnected cell assemblies. By studying the dynamics of *in vitro* networks (see Figures 2, 3), we will create a computational model (see Figure 6) exhibiting the same I/O function of its biological counterpart (Figure 8, panel A). Through this approach we also plan to further our knowledge about the interplay between structural connectivity and dynamics in neuronal networks. Once we have realized and tested our model, we will bi-directionally integrate it into a biological network made up of few interconnected sub-networks in replacement of one of these that has been previously lesioned (Figure 8A, right panel).

The same conceptual approach will be applied to the olfactory-limbic pathway in the IWB (Figure 8, panel B). After a thorough characterization of spontaneous activity patterns (e.g., spontaneous periodic events, which strikingly resemble the ones shown by primary cortical cultures; see Figure 1) and LOT-evoked responses generated in the m-ERC (see Figure 8), we will include such information into a realistic computational model. We will then induce an ischemic lesion of the hippocampus and realize a functional model able to reproduce the same transfer function of the damaged part in order to restore the original pathway. Figure 8 summarizes this approach, both for *in vitro* interconnected finite-size networks and for the guinea pig IWB.

**Figure 9:**
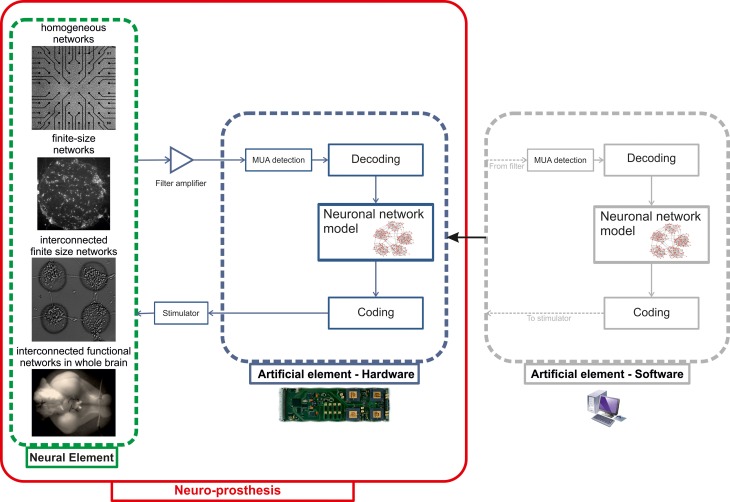
**Schematic representation of the closed-loop system to be implemented as a proof-of-concept neuro-prosthesis.** Different *in vitro* neural models with increasing degrees of architectural complexity (“Neural element”) will be interfaced to a hardware neuromorphic chip (“Artificial element—Hardware”), implementing both signal processing (“MUA detection”) and modeling (“Neuronal network model”) algorithms previously tested in software (“Artificial element—Software”). The communication between the neuronal network model and its biological counterpart is accomplished by the “Coding” and “Decoding” blocks.

The final step foreseen in the BRAIN BOW project is the hardware implementation of the signal processing algorithms and computational models to achieve our proof-of-concept neuroprosthesis based on a neuromorphic chip. Figure 9 illustrates the closed-loop architecture that we plan to develop. Raw traces recorded by means of either planar or implanted MEAs (depending on the biological sample) will be fed into the artificial element and pre-processed online to extract multi-unit activity patterns (MUA). Spatio-temporal spiking patterns will then be translated by the “decoding” block into signals delivered to the neuronal network model. After elaboration, output patterns produced by the model will be finally translated by the “coding” block into a stimulation delivered to the neural element (Figure 9).

## Discussion

This paper presents a bottom-up, multidisciplinary approach toward the realization of a neural prosthesis capable of replacing lesioned neuronal circuitries. The final goal of the studies consists of developing a neuromorphic chip reproducing the function of a lesioned circuit without replicating its specific architecture or structural organization.

As a general model of a self-organized neuronal circuit, finite size neuronal circuits in culture are produced and studied in an isolated configuration to reveal innate (and therefore most general) features of intra-circuit organization (cf. Figure 1). Since finite size networks can spontaneously interconnect in a “multi-modular” network organization, they also represent an optimal experimental model to reveal innate inter-circuit communication properties (cf. Figure 1), as shown in previous studies (Macis et al., 2007; Raichman and Ben-Jacob, 2008; Shein-Idelson et al., 2010, 2011). The structural—functional configuration of the finite size circuits can be replicated by an *in silico* neuronal network and then implemented on a neuromorphic prosthetic chip. The capability of the neuromorphic chip to replace the function of a lesioned circuit will be tested at increasing levels of network complexity from an *in vitro* modular network to an isolated whole brain system (IWB). In the attempt to present the overall scientific approach of the BRAIN BOW project (cf. section “Introduction” and “A Bi-Directional Neuro-Prosthesis”), this paper shows first results from the different level of investigation grounding the overall strategy.

### Finite size circuits and innate functional organization of cortical circuits

As previously shown by Shein-Idelson et al. (2010), cultured cortical neuronal networks composed of at least a few dozen neurons are able to produce spontaneous collective dynamics known as network bursts, characterized by oscillatory activity in the gamma-theta range, and with the frequency of the bursts increasing with the number of neurons in the network. We confirmed these findings here using optical measurements on monolayer circuits (cf. Figure 2). By combining calcium imaging with immunocytochemistry, we have found that network events first recruit a characteristic population of neurons which includes GABAergic neurons. In particular, the time-lag correlation of the finite size cortical circuits is similar to what observed in developing hippocampal circuits (Bonifazi et al., 2009), in which a scale-free functional connectivity distribution was accompanied by the existence of GABAergic hub cells able to play a key role in the orchestration of the spontaneous network events. All together, these observations suggest that cortical neuronal circuits share a common innate functional organization which might include the existence of GABAergic hub cells.

### Monitoring effects of lesioned neuronal circuits in finite size networks

After characterizing the spontaneous dynamics of the finite size networks we monitored how a focalized lesion can trigger functional reorganization in the neuronal circuit. We made controlled laser ablations of different intensities on our networks (e.g., targeting single modules, inter-connections between modules, single neuritis/cell bodies/cell assembly). After the lesions, the neuronal circuits continued to produce spontaneous networks events with no significant changes in the frequency of occurrence (Figures 3 and A1). These were presumably generated out of the imaged field where the lesions were performed. The number of cells recruited during network events decreased either because they were directly lesioned by the laser ablation or because of a change in the local functional organization of the circuits (see the functional connectivity graphs of Figure 4). In a previous study by Maeda et al. (1995) the authors made a lesion in a homogeneous network over a MEA to study the origin of spontaneous network bursting. More recently, Difato et al. (2011a) reported controlled sequential ablation of single connections in a neuronal network, causing modulation of its activity without irreversibly damaging it. By combining MEA recording and calcium imaging the authors found changes in electrophysiological patterns in the network and identified the contribution of neuronal sub-populations to the network activity (Difato et al., 2011b). To the best of our knowledge, our study is the first to make a spatially defined micro-lesion at the single cell scale and to analyze the neuronal dynamics and connectivity by means of optical-only tools. This methodology, which can be extended to the use of genetically encoded calcium sensors, allows a more detailed and prolonged monitoring of the functional reorganization of the circuit over hours or days with the advantage, when compared to electrophysiological recordings, that the high spatial resolution (i.e., single cell) can be linked to morphological/structural cellular properties through *post-hoc* immunocytochemical characterizations. This could also facilitate testing of methods to promote functional circuit repair, such as pharmacological approaches.

### Simulation results (software and hardware)

Given the similarity between the synchronization dynamics observed in developing hippocampal networks (Bonifazi et al., 2009) and in the finite circuits (Figure 2) with early activated GABAergic cells forecasting synchrony, we hypothesized a common innate structural-functional organization in neocortical and paleocortical circuits. Therefore, we used a scale-free topology (Barabasi and Albert, 1999) to model a neuronal network based on Izhikevich neurons (Izhikevich, 2003) (Figure 6). The proposed model was able to reproduce the empirical dependence between bursting rate and circuit size. However, the model predicted a richer repertoire of firing patterns (e.g., Figure 6B). Indeed, such patterns can be found in biological networks (Segev et al., 2002; Macis et al., 2007; Marconi et al., 2012). Thus our synthetic models (conveniently tuned and adapted) are able to reproduce the dynamics found in *in vitro* networks. Our results also demonstrate that the hardware element of the prosthesis (cf. section “Hardware Set-up for a Brain Prosthesis” and “A Bi-Directional Neuro-Prosthesis”) can be constituted by a neuromorphic model (SNN) built on the same equations as the computational model (Izhikevich, 2003), since it reproduces similar firing rate distributions (Figure 7). Thus, the computational (software) model serves as a bridge between the biological networks and the hardware implementation.

### Comparison to previous work and prospective results

In the last decades, great efforts have been made to develop neuro-prostheses to restore lost sensory or motor functions (Taylor et al., 2002; Chader et al., 2009; Collinger et al., 2013), but very few groups have focused on neuro-prostheses targeting lesions at the level of the CNS and aimed at recovering lost cognitive capabilities (Berger et al., 2011; Prueckl et al., 2011; Bamford et al., 2012; Hampson et al., 2012; Opris et al., 2012). Although our studies are limited to simplified *in vitro* models of cell assemblies, their final aim is to provide useful insights for the design of future cognitive prostheses. We believe that our approach would help us understand how we can influence/drive the dynamics of a neuronal assembly by interfacing it to an artificial network, implemented either in software or hardware. This is not the first attempt to realize an *in vitro* closed-loop system: previous studies have used a robotic actuator or a control algorithm aimed at clamping network activity to a desired level (Demarse et al., 2001; Martinoia et al., 2004; Wagenaar et al., 2005; Wallach et al., 2011). However, we seek to extend these approaches by replacing a real biological network with a simulated network, and hence by implementing bi-directional communication between biological and simulated networks. This research project builds on previously published results in the field of *in vitro* closed-loop electrophysiology (Arsiero et al., 2007). It can also be generalized to a more structured experimental model like the *in vitro* whole brain of a guinea pig, which lies between *in vivo* (as it retains the original tridimensional architecture) and *in vitro* (as it is disconnected from any sensory input/motor output). In contrast to other groups which have exclusively investigated *in vivo* brain prostheses (Prueckl et al., 2011; Bamford et al., 2012; Berger et al., 2012; Hampson et al., 2012; Opris et al., 2012), we are trying to exploit the unique advantages of *in vitro* electrophysiology—accessibility, visibility and control of physical and chemical conditions—to study neural information processing in neuronal assemblies, and to understand which parameters are relevant for effectively interfacing biological and artificial networks. In addition to informing the design of future *in vivo* approaches, our approach could also illuminate how network structure constrains and drives network dynamics.

### Conflict of interest statement

The authors declare that the research was conducted in the absence of any commercial or financial relationships that could be construed as a potential conflict of interest.

## References

[B1] Arsiero M. Luscher H. R. Giugliano M. (2007). Real-time closed-loop electrophysiology: towards new frontiers in *in vitro* investigations in the neurosciences. Arch. Ital. Biol. 145, 193–209 18075116

[B2] Bamford S. A. Hogri R. Giovannucci A. Taub A. H. Herreros I. Verschure P. F. (2012). A VLSI field-programmable mixed-signal array to perform neural signal processing and neural modeling in a prosthetic system. IEEE Trans. Neural Syst. Rehabil. Eng. 20, 455–467 10.1109/TNSRE.2012.2187933 22481832

[B3] Barabasi A.-L. Albert R. (1999). Emergence of scaling in random networks. Science 286, 509–512 10.1126/science.286.5439.509 10521342

[B4] Batagelj V. Brandes U. (2005). Efficient generation of large random networks. Phys. Rev. E 71, 036113 15903499 10.1103/PhysRevE.71.036113

[B5] Berger T. W. Hampson R. E. Song D. Goonawardena A. Marmarelis V. Z. Deadwyler S. A. (2011). A cortical neural prosthesis for restoring and enhancing memory. J. Neural Eng. 8:046017 10.1088/1741-2560/8/4/046017 21677369 PMC3141091

[B6] Berger T. W. Song D. Chan R. H. Marmarelis V. Z. Lacoss J. Wills J. (2012). A hippocampal cognitive prosthesis: multi-input, multi-output nonlinear modeling and VLSI implementation. IEEE Trans. Neural Syst. Rehabil. Eng. 20, 198–211 10.1109/TNSRE.2012.2189133 22438335 PMC3395724

[B7] Biella G. De Curtis M. (2000). Olfactory inputs activate the medial entorhinal cortex via the hippocampus. J. Neurophysiol. 83, 1924–1931 10758103 10.1152/jn.2000.83.4.1924

[B8] Bologna L. L. Pasquale V. Garofalo M. Gandolfo M. Baljon P. L. Maccione A. (2010). Investigating neuronal activity by SPYCODE multichannel data analyzer. Neural Netw. 23, 685–697 10.1016/j.neunet.2010.05.002 20554151

[B9] Bonifazi P. Goldin M. Picardo M. A. Jorquera I. Cattani A. Bianconi G. (2009). GABAergic hub neurons orchestrate synchrony in developing hippocampal networks. Science 326, 1419–1424 10.1126/science.1175509 19965761

[B11a] Canto C. B. Wouterlood F. G. Witter M. P. (2008). What does the anatomical organization of the entorhinal cortex tell us? Neural. Plast. 2008:381243 10.1155/2008/381243 18769556 PMC2526269

[B10] Cassidy A. Andreou A. G. (2008). Dynamical digital silicon neurons, in Biomedical Circuits and Systems Conference, 2008. BioCAS (2008). IEEE (Baltimore, MD).

[B11] Chader G. J. Weiland J. Humayun M. S. (2009). Artificial vision: needs, functioning, and testing of a retinal electronic prosthesis. Prog. Brain Res. 175, 317–332 10.1016/S0079-6123(09)17522-2 19660665

[B12] Chiappalone M. Bove M. Vato A. Tedesco M. Martinoia S. (2006). Dissociated cortical networks show spontaneously correlated activity patterns during *in vitro* development. Brain Res. 1093, 41–53 10.1016/j.brainres.2006.03.049 16712817

[B13] Collinger J. L. Wodlinger B. Downey J. E. Wang W. Tyler-Kabara E. C. Weber D. J. (2013). High-performance neuroprosthetic control by an individual with tetraplegia. Lancet 381, 557–564 10.1016/S0140-6736(12)61816-9 23253623 PMC3641862

[B14] Crépel V. Aronov D. Jorquera I. Represa A. Ben-Ari Y. Cossart R. (2007). A parturition-associated nonsynaptic coherent activity pattern in the developing hippocampus. Neuron 54, 105–120 10.1016/j.neuron.2007.03.007 17408581

[B15] De Curtis M. Biella G. Buccellati C. Folco G. (1998). Simultaneous investigation of the neuronal and vascular compartments in the guinea pig brain isolated *in vitro*. Brain Res. Brain Res. Protoc. 3, 221–228 10.1016/S1385-299X(98)00044-0 9813340

[B16] Demarse T. B. Wagenaar D. A. Blau A. W. Potter S. M. (2001). The neurally controlled animat: biological brains acting with simulated bodies. Auton. Robots 11, 305–310 18584059 10.1023/a:1012407611130 PMC2440704

[B17] Difato F. Dal Maschio M. Marconi E. Ronzitti G. Maccione A. Fellin T. (2011a). Combined optical tweezers and laser dissector for controlled ablation of functional connections in neural networks. J. Biomed. Opt. 16:051306 10.1117/1.3560268 21639566

[B18] Difato F. Schibalsky L. Benfenati F. Blau A. (2011b). Integration of optical manipulation and electrophysiological tools to modulate and record activity in neural networks. Int. J. Optomechatronic. 5, 191–216

[B19] Dorogovtsev S. Mendes J. (2002). Evolution of networks. Adv. Phys. 51, 1079–1187

[B20] Eytan D. Marom S. (2006). Dynamics and effective topology underlying synchronization in networks of cortical neurons. J. Neurosci. 26, 8465–8476 10.1523/JNEUROSCI.1627-06.2006 16914671 PMC6674346

[B21] Gnatkovsky V. De Curtis M. (2006). Hippocampus-mediated activation of superficial and deep layer neurons in the medial entorhinal cortex of the isolated guinea pig brain. J. Neurosci. 26, 873–881 10.1523/JNEUROSCI.4365-05.2006 16421307 PMC6675375

[B22] Hampson R. E. Gerhardt G. A. Marmarelis V. Song D. Opris I. Santos L. (2012). Facilitation and restoration of cognitive function in primate prefrontal cortex by a neuroprosthesis that utilizes minicolumn-specific neural firing. J. Neural Eng. 9:056012 10.1088/1741-2560/9/5/056012 22976769 PMC3505670

[B23] Herzog N. Shein-Idelson M. Hanein Y. J. (2011). Optical validation of *in vitro* extra-cellular neuronal recordings. J. Neural Eng. 8:056008 10.1088/1741-2560/8/5/056008 21841241

[B24] Hochberg L. R. Bacher D. Jarosiewicz B. Masse N. Y. Simeral J. D. Vogel J. (2012). Reach and grasp by people with tetraplegia using a neurally controlled robotic arm. Nat. Methods 485, 372–375 10.1038/nature11076 22596161 PMC3640850

[B25] Hochberg L. R. Serruya M. D. Friehs G. M. Mukand J. A. Saleh M. Caplan A. H. (2006). Neuronal ensemble control of prosthetic devices by a human with tetraplegia. Nature 442, 164–171 10.1038/nature04970 16838014

[B26] Isaacson J. S. Scanziani M. (2011). How inhibition shapes cortical activity. Neuron 72, 231–243 10.1016/j.neuron.2011.09.027 22017986 PMC3236361

[B27] Izhikevich E. M. (2003). Simple model of spiking neurons. IEEE Trans. Neural Netw. 6, 1569–1572 10.1109/TNN.2003.820440 18244602

[B28] Kamioka H. Maeda E. Jimbo Y. Robinson H. P. Kawana A. (1996). Spontaneous periodic synchronized bursting during formation of mature patterns of connections in cortical cultures. Neurosci. Lett. 206, 109–112 8710163 10.1016/s0304-3940(96)12448-4

[B29] Kleinfeld D. Kahler K. H. Hockberger P. E. (1988). Controlled outgrowth of dissociated neurons on patterned substrates. J. Neurosci. 8, 4098–4120 3054009 10.1523/JNEUROSCI.08-11-04098.1988 PMC6569488

[B30] Librizzi L. Biella G. Cimino C. De Curtis M. (1999). Arterial supply of limbic structures in the guinea pig. J. Comp. Neurol. 4, 674–682 10.1002/(SICI)1096-9861(19990906)411:4<674::AID-CNE11>3.0.CO;2-O 10421876

[B31] Llinas R. Yarom Y. Sugimori M. (1981). Isolated mammalian brain *in vitro*: new technique for analysis of electrical activity of neuronal circuit function. Fed. Proc. 40, 2240–2245 7238908

[B32] Maccione A. Gandolfo M. Massobrio P. Novellino A. Martinoia S. Chiappalone M. (2009). A novel algorithm for precise identification of spikes in extracellularly recorded neuronal signals. J. Neurosci. Methods 177, 241–249 10.1016/j.jneumeth.2008.09.026 18957306

[B33] Macis E. Tedesco M. Massobrio P. Raiteri R. Martinoia S. (2007). An automated microdrop delivery system for neuronal network patterning on microelectrode arrays. J. Neurosci. Methods 161, 88–95 10.1016/j.jneumeth.2006.10.015 17141327

[B34] Maeda E. Robinson H. P. Kawana A. (1995). The mechanisms of generation and propagation of synchronized bursting in developing networks of cortical neurons. J. Neurosci. 15, 6834–6845 7472441 10.1523/JNEUROSCI.15-10-06834.1995 PMC6578010

[B35] Marconi E. Nieus T. Maccione A. Valente P. Simi A. Messa M. (2012). Emergent functional properties of neuronal networks with controlled topology. PLoS ONE 7:e34648 10.1371/journal.pone.0034648 22493706 PMC3321036

[B36] Marissal T. Bonifazi P. Picardo M. A. Nardou R. Petit L. F. Baude A. (2012). Pioneer glutamatergic cells develop into a morpho-functionally distinct population in the juvenile CA3 hippocampus. Nat. Commun. 3, 1316 10.1038/ncomms2318 23271650 PMC3535425

[B37] Martinoia S. Sanguineti V. Cozzi L. Berdondini L. Van Pelt J. Tomas J. (2004). Towards an embodied *in-vitro* electrophysiology: the NeuroBIT project. Neurocomputing 58–60, 1065–1072

[B38] Muhlethaler M. De Curtis M. Walton K. Llinas R. (1993). The isolated and perfused brain of the guinea-pig *in vitro*. Eur. J. Neurosci. 5, 915–926 8281302 10.1111/j.1460-9568.1993.tb00942.x

[B39] Nicolelis M. A. L. (2003). Brain-Machine interfaces to restore motor function and probe neural circuits. Nat. Rev. Neurosci. 4, 417–422 10.1038/nrn1105 12728268

[B40] Nicolelis M. A. L. Lebedev M. A. (2009). Principles of neural ensemble physiology underlying the operation of brain-machine interfaces. Nat. Rev. Neurosci. 10, 530–540 10.1038/nrn2653 19543222

[B41] Novellino A. D'Angelo P. Cozzi L. Chiappalone M. Sanguineti V. Martinoia S. (2007). Connecting neurons to a mobile robot: an *in vitro* bidirectional neural interface. Comput. Intell. Neurosci. 2007:12725 10.1155/2007/12725 18350128 PMC2266971

[B42] Opris I. Fuqua J. L. Huettl P. F. Gerhardt G. A. Berger T. W. Hampson R. E. (2012). Closing the loop in primate prefrontal cortex: inter-laminar processing. Front. Neural Circuits 6:88 10.3389/fncir.2012.00088 23189041 PMC3504312

[B43] Pastori C. Regondi M. C. Librizzi L. De Curtis M. (2007). Early excitability changes in a novel acute model of transient focal ischemia-reperfusion in the *in vitro* isolated guinea pig brain. Exp. Neurol. 204, 95–105 10.1016/j.expneurol.2006.09.023 17141221

[B44] Perkel D. H. Gerstein G. L. Moore G. P. (1967). Neuronal spike trains and stochastic point processes. I. The single spike train. Biophys. J. 7, 391–418 10.1016/S0006-3495(67)86596-2 4292791 PMC1368068

[B45] Prueckl R. Taub A. H. Herreros I. Hogri R. Magal A. Bamford S. A. (2011). Behavioral rehabilitation of the eye closure reflex in senescent rats using a real-time biosignal acquisition system, in Engineering in Medicine and Biology Society, EMBC (2011). (Boston, MA). 10.1109/IEMBS.2011.6091045 22255268

[B46] Raichman N. Ben-Jacob E. (2008). Identifying repeating motifs in the activation of synchronized bursts in cultured neuronal networks. J. Neurosci. Methods 170, 96–110 10.1016/j.jneumeth.2007.12.020 18281097

[B47] Raoux M. Bornat Y. Quotb A. Catargi B. Renaud S. Lang J. (2012). Non-invasive long-term and real-time analysis of endocrine cells on micro-electrode arrays. J. Physiol. 590, 1085–1091 10.1113/jphysiol.2011.220038 22199167 PMC3381817

[B48] Rieke F. Warland D. De Ruyter Van Steveninck R. Bialek W. (1997). Spikes: Exploring the Neural Code. Cambridge, MA: The MIT Press

[B49] Segev R. Benveniste M. Hulata E. Cohen N. Palevski A. (2002). Long term behavior of lithographically prepared *in vitro* neuronal networks. Phys. Rev. Lett. 88, 1181021–1118104 10.1103/PhysRevLett.88.118102 11909430

[B50] Shein-Idelson M. Ben-Jacob E. Hanein Y. (2010). Innate synchronous oscillations in freely-organized small neuronal circuits. PLoS ONE 5:e14443 10.1371/journal.pone.0014443 21203438 PMC3010988

[B51] Shein-Idelson M. Ben-Jacob E. Hanein Y. (2011). Engineered neuronal circuits: a new platform for studying the role of modular topology. Front. Neuroeng. 4:10 10.3389/fneng.2011.00010 21991254 PMC3180629

[B52] Sorkin R. Gabay T. Blinder P. Baranes D. Ben-Jacob E. Hanein Y. (2006). Compact self-wiring in cultured neural networks. J. Neural Eng. 3, 95–101 10.1088/1741-2560/3/2/003 16705265

[B53] Takano H. Mccartney M. Ortinski P. I. Yue C. Putt M. E. Coulter D. A. (2012). Deterministic and stochastic neuronal contributions to distinct synchronous CA3 network bursts. J. Neurosci. 32, 4743–4754 10.1523/JNEUROSCI.4277-11.2012 22492030 PMC3328771

[B54] Taylor D. M. Tillery S. I. Schwartz A. B. (2002). Direct cortical control of 3D neuroprosthetic devices. Science 296, 1829–1832 10.1126/science.1070291 12052948

[B55] Tessadori J. Bisio M. Martinoia S. Chiappalone M. (2012). Modular neuronal assemblies embodied in a closed-loop environment: toward future integration of brains and machines. Front. Neural Circuits 6:99 10.3389/fncir.2012.00099 23248586 PMC3520178

[B56] Van Pelt J. Wolters P. S. Corner M. A. Rutten W. L. Ramakers G. J. (2004). Long-term characterization of firing dynamics of spontaneous bursts in cultured neural networks. IEEE Trans. Biomed. Eng. 51, 2051–2062 10.1109/TBME.2004.827936 15536907

[B57] Wagenaar D. A. Madhavan R. Pine J. Potter S. M. (2005). Controlling bursting in cortical cultures with closed-loop multi-electrode stimulation. J. Neurosci. 25, 680–688 10.1523/JNEUROSCI.4209-04.2005 15659605 PMC2663856

[B58] Wagenaar D. A. Potter S. M. (2002). Real-time multi-channel stimulus artifact suppression by local curve fitting. J. Neurosci. Methods 120, 113–120 10.1016/S0165-0270(02)00149-8 12385761

[B59] Wallach A. Eytan D. Gal A. Zrenner C. Marom S. (2011). Neuronal response clamp. Front. Neuroeng. 4:3 10.3389/fneng.2011.00003 21519391 PMC3078750

